# Hyperbaric Oxygen Therapy for Vascular Air Embolism From Iatrogenic Intravenous Infusion of Air in a Patient With Atrial Septal Defect: A Case Report

**DOI:** 10.7759/cureus.9554

**Published:** 2020-08-04

**Authors:** Julian S Trent, Joni K Hodgson, Bret Ackermann, Nicholas M Studer

**Affiliations:** 1 Department of Emergency Medicine, San Antonio Uniformed Services Health Education Consortium, San Antonio, USA; 2 Undersea and Hyperbaric Medicine, San Antonio Uniformed Services Health Education Consortium, San Antonio, USA

**Keywords:** hyperbaric medicine, hyperbaric oxygen, vascular air embolism, arterial gas embolism, venous gas embolism, emergency medicine, atrial septal defect

## Abstract

Vascular air embolism (VAE) is an important complication of some routine medical procedures, particularly intravenous access for the administration of fluids or medications. The capillary bed of the pulmonary circulatory system is capable of compensating for small amounts of air entrained into a vein. However, large amounts of air can overwhelm that system and lead to complications ranging from cough, chest pain, or shortness of breath to cardiopulmonary collapse. Additionally, air entrained directly into the arterial system, or that which crosses from the venous system to the arterial system through a shunt can cause the acute coronary syndrome, loss of consciousness, arrhythmias, altered mental status, stroke, or limb ischemia. We present a case in which a patient with a known atrial septal defect had a moderate volume of air entrained through an intravenous catheter requiring hyperbaric oxygen therapy.

## Introduction

Iatrogenic vascular air embolism (VAE) is a life-threatening emergency that can occur during a variety of routine medical and surgical procedures. Venous gas emboli (VGE) can lead to cough, pleuritic chest pain, hypoxic respiratory failure, and hemodynamic collapse. Most frequently, these emboli are introduced through peripheral or central venous catheters during the administration of fluids or during procedures that expose the venous blood to a gas. Arterial gas emboli (AGE) can obstruct both cerebrovascular and coronary circulation leading to stroke and myocardial ischemia, respectively. Arterial emboli are most commonly introduced through barotrauma from mechanical ventilation, during surgeries involving the cardiopulmonary systems, or during arterial line placement [1]. Paradoxical gas emboli are entrained in the venous system, but cross over to the arterial system through a patent foramen ovale, septal defect, or intrapulmonary arteriovenous anastomosis.

## Case presentation

A 33-year-old male with a known four-millimeter atrial septal defect who worked as a senior medical technician in a military ED had an intravenous catheter placed by one of his trainees in his left antecubital vein. After placement of the intravenous line, a 1-liter bag of Lactated Ringer’s solution was rapidly infused via pressure bag. After the infusion of Lactated Ringer’s solution, an unknown quantity of air was inadvertently infused into the patient. He immediately developed pleuritic chest discomfort and cough. His initial vital signs were within normal limits, with the exception of mild tachycardia to 103 beats per minute. On physical exam, no murmur was auscultated, his lungs were clear to auscultation bilaterally; he had no relevant skin findings, and no neurological deficits. He was placed in the Trendelenburg position and supplemental oxygen was administered at a rate of 15 liters per minute via non-rebreather mask. With these interventions, the patient’s symptoms improved significantly. A chest X-ray was performed with no acute cardiopulmonary abnormalities. CT pulmonary angiogram was obtained without visualization of air in the pulmonary vasculature or aerocardia. 

The Undersea and Hyperbaric Medicine Service was consulted for evaluation and treatment with hyperbaric oxygen. After being transferred to the Undersea and Hyperbaric Medicine Clinic, the patient removed his supplemental oxygen and stood up to ambulate to the restroom, which resulted in return of his symptoms. The patient was unable to tolerate the increasing pressure in the hyperbaric chamber initially due to the inability to successfully clear his ears. Oxymetazoline nasal spray was administered, and the patient was able to reach treatment depth of 3.0 atmospheres absolute (ATA) (66 feet of saltwater [fsw]) for 30 minutes, followed by 60 minutes at 2.5 ATA (49.5 fsw). He was then returned to ambient pressure for a total time at depth of 120 minutes, in accordance with the monoplace treatment table originally described by Hart et al. [2]. After treatment, he was transported back to the ED. Upon reassessment, the patient had complete resolution of symptoms, and was discharged from the ED. On follow-up with the patient two days later, the patient denied the interim return of his cough or chest discomfort.

## Discussion

Symptoms of VAE are proportional to the volume and rate of entrainment of air into the vasculature. The lethal volume of intravascular air in humans is estimated to be approximately 3-5 cc/kg [3]. The pulmonary capillary bed is capable of filtering an estimated 0.3 cc/kg/min according to animal models [4]. When this filtration capability is exceeded, symptoms develop depending on the remaining volume of air. Small volumes (<0.5 cc/kg) lead to wheezing, minor decreases in oxygen saturation and end-tidal carbon dioxide, or an increase in end-tidal nitrogen levels. Moderate volumes (0.5-2.0 cc/kg) can produce hypotension, bronchoconstriction, cerebral or myocardial ischemia, altered mental status, and respiratory distress. Large quantities (>2.0 cc/kg) can result in cardiovascular collapse, respiratory failure, and death [1].

The diagnosis of VAE requires high clinical suspicion in settings where entrainment of air is possible. Onset of symptoms is often rapid. Sudden changes in mental status, cardiac tracings, and respiratory or hemodynamic monitoring during vascular access or surgical procedures with high air embolism risk should be highly suspicious for VAE. Surgical procedures considered high risk for VAE include craniotomy, neck surgery, laparoscopic procedure, coronary artery bypass grafting, upper and lower endoscopy, hip arthroplasty, lung biopsy, and Cesarean section [5,6]. Detection of air in the cardiovascular system can prove challenging. Transesophageal echocardiography is considered the most sensitive imaging modality; however, its practicality is limited in the ED given its invasiveness and requirement for specialty training [7]. Precordial Doppler is another sensitive screening tool frequently used by anesthesiologists during high-risk procedures for early detection of as little as 0.25 mL of air [8]. While many case reports discuss the use of CT for the diagnosis of VAE, it is not sensitive enough to rule-out the diagnosis [1]. AGE can cause an increase in pulmonary artery pressure measured with pulmonary artery catheter; however, the sensitivity is only 15% [7]. If clinical suspicion is strong for VAE versus other potential etiologies, imaging should be deferred if it will delay definitive treatment [9].

The first step in management of VAE is to prevent continued air infusion. During surgical procedures, this may involve covering the surgical field with saline-soaked dressing to create a barrier between the atmosphere and a blood vessel that has been compromised. Stopping intravenous infusions of air by shutting off a pump or clamping a port is an important first step. Administration of supplemental oxygen with a fraction of inspired oxygen as close to 100% as possible aids in the elimination of nitrogen, thereby decreasing the size of the embolism [10]. It also aids in oxygenation, which may be compromised as a direct result of the emboli. Continue supportive care of hemodynamic instability with fluid resuscitation, vasopressors, and/or inotropic agents as necessary based on clinical presentation. Patient positioning is a controversial component of VAE management. Traditional left lateral decubitus positioning has been shown to relocate intracardiac air on transesophageal echocardiography; however, it has not been shown to improve hemodynamics [11]. Animal models have failed to demonstrate improved hemodynamics with Trendelenburg positioning, and in cases of arterial air embolism, it should be avoided as it may worsen cerebral edema [12]. It has been suggested that the primary mechanism of cardiac dysfunction in venous air embolism arises from right ventricular outflow obstruction by large emboli, referred to as vapor lock [7]. In cases of vapor lock, the Durant Maneuver, whereby the patient is placed in the left lateral decubitus and Trendelenburg position, may be effective in shifting the gas embolism enough to allow for right ventricular outflow. While aspiration of air from the right atrium via Swan-Ganz catheter is a proposed treatment modality, numerous animal studies have demonstrated a lack of efficacy [1]. However, during cardiovascular collapse, it may be the only last-ditch effort while CPR is ongoing. There are a few studies showing slightly more optimistic results in dogs with hemodynamic collapse secondary to air gas embolism using a Bunegin-Albin catheter [13]. Hyperbaric oxygen therapy decreases the size of gas emboli in two ways. First, by increasing the pressure exerted on a gas bubble in a liquid, its volume decreases as described by Boyle’s Law [9]. Secondly, by increasing the partial pressure of oxygen in the blood, more nitrogen leaves the bubble into the bloodstream or adjacent tissues due to the increased oxygen diffusion gradient described by Henry’s Law [9]. Nitrogen dissolved in the blood is then removed during exhalation [7]. Hyperbaric oxygen therapy is most effective when initiated within the first 6 hours after insult [3], and is effective for both arterial and venous emboli [14,15]. In addition to shrinking bubbles, hyperbaric oxygen therapy decreases cerebral edema by vascular vasoconstriction and decreases reperfusion injury and inflammation by inhibiting neutrophil endothelial adhesion [9].

Monoplace treatment tables, similar to the one utilized for this patient, do not incorporate air breaks in the treatment table. Therefore, they are usually shorter than treatment tables that do utilize air breaks. Shorter, monoplace treatment tables are appropriate if symptoms resolve within the first 10 minutes of treatment at depth, otherwise a longer treatment table with air breaks should be used. Return of symptoms is an indication for repeat treatment, but patients that underwent a monoplace treatment table should be given an air break, breathing air at 1 ATA for at least 30 minutes [9].

## Conclusions

Early recognition and management of VAE can significantly impact the high mortality of this life-threatening consequence of common medical procedures. VAE should be considered when changes in respiratory status or hemodynamics occur in scenarios where air may have entered the cardiovascular system. Management with supportive care and supplemental oxygen should begin immediately. Patient positioning is controversial, but Durant’s maneuver may be beneficial in cases of vapor lock. Hyperbaric oxygen therapy is an effective way to decrease emboli size, decrease reperfusion injury, and increase the rate at which gas bubbles are removed by the lungs.

## References

[1] Mirski MA, Lele AV, Fitzsimmons L, Toung TJK (2007). Diagnosis and treatment of vascular air embolism. Anesthesiol J Am Soc Anesthesiol.

[2] Hart G, Strauss M, Lennon P (1986). The treatment of decompression sickness and air embolism in a monoplace chamber. J Hyperb Med.

[3] McCarthy CJ, Behravesh S, Naidu SG, Oklu R (2017). Air embolism: diagnosis, clinical management and outcomes. Diagnostics.

[4] Butler BD, Hills BA (1985). Transpulmonary passage of venous air emboli. J Appl Physiol.

[5] McCarthy CJ, Behravesh S, Naidu SG, Oklu R (2016). Air embolism: practical tips for prevention and treatment. J Clin Med.

[6] Malik N, Claus PL, Illman JE (2017). Air embolism: diagnosis and management. Future Cardiol.

[7] Shaikh N, Ummunisa F (2009). Acute management of vascular air embolism. J Emerg Trauma Shock.

[8] Schubert A, Deogaonkar A, Drummond JC (2006). Precordial Doppler probe placement for optimal detection of venous air embolism during craniotomy. Anesth Analg.

[9] Neuman TS, Thom SRe (2008). Physiology and Medicine of Hyperbaric Oxygen Therapy.

[10] van Hulst RA, Klein J, Lachmann B (2003). Gas embolism: pathophysiology and treatment. Clin Physiol Funct Imaging.

[11] Geissler HJ, Allen SJ, Mehlhorn U, Davis KL, Morris WP, Butler BD (1997). Effect of body repositioning after venous air embolism: an echocardiographic study. Anesthesiology.

[12] Mehlhorn U, Burke EJ, Butler BD (1994). Body position does not affect the hemodynamic response to venous air embolism in dogs. Anesth Analg.

[13] Colley PS, Artru AA (1989). Bunegin-Albin catheter improves air retrieval and resuscitation from lethal venous air embolism in upright dogs. Anesth Analg.

[14] Scruggs JE, Joffe A, Wood KE (2008). Paradoxical air embolism successfully treated with hyperbaric oxygen. J Intensive Care Med.

[15] Torres Martinez FJ, Kuffler DP (2011). Hyperbaric oxygen treatment to eliminate a large venous air embolism: a case study. Undersea Hyperb Med.

